# Development of job demands, decision authority and social support in industries with different gender composition – Sweden, 1991–2013

**DOI:** 10.1186/s12889-019-6917-8

**Published:** 2019-06-14

**Authors:** Sara Cerdas, Annika Härenstam, Gun Johansson, Anna Nyberg

**Affiliations:** 1 Stress Research Institute, Stockholm University, SE-106 91 Stockholm, Sweden; 2 Department of Psychology, Stockholm University, SE-106 91 Stockholm, Sweden; 3 Institute of Environmental Medicine, Karolinska Institutet, SE-113 65 Stockholm, Sweden

**Keywords:** Job demands, Decision authority, Social support, Sweden, Gender composition, Industry, Development over time, Average marginal effects

## Abstract

**Background:**

This study aims to explore the development of job demands, decision authority and social support within and between industries with different gender composition in Sweden between 1991 and 2013.

**Methods:**

Cross-sectional data from 12 waves of the Swedish Work Environment Surveys (1991 to 2013), comprising in total 109,698 respondents, were used. Industries were classified in 7 categories according to its gender composition and main activity, comprising two female-dominated, three gender-mixed and two male-dominated industries. Proportions of workers reporting high job demands, low decision authority and poor social support between 1991 and 2013 were calculated. Logistic regression analyses were performed to estimate variation across time, using 1991 as the reference category, and between industries, using knowledge intensive services as the reference category. Estimates for high job demands, low decision authority and poor social support were presented as average marginal effects (AMEs).

**Results:**

The probabilities of reporting low decision authority were higher in education and health and social care during the whole study period, for both genders, compared with the reference category of knowledge intensive services. The probability of having high job demands were higher for men and women in education, and women in health and social care, compared with the reference category. Men in the male dominated industries had increased job demands over time, compared to the beginning of the study period (1991). The probability of reporting poor social support was higher in the later than in the earliest time period for women in the female-dominated industry health and social care as well as in the gender-mixed labour intensive services industry.

**Conclusions:**

There has been a negative development of job demands and decision authority in the female-dominated industries education and health and social care in Sweden, whereas social support has developed more negatively for women in health and social care and in labour intensive services.

**Electronic supplementary material:**

The online version of this article (10.1186/s12889-019-6917-8) contains supplementary material, which is available to authorized users.

## Background

Sickness absence for psychiatric disorders has been increasing in Sweden in the last decades, mainly due to stress-related and depressive disorders, and are currently the most common causes of sick leave [1]. The negative development has been particularly pronounced among employees in female-dominated industries, such as health and social care and education, and the highest rates are found among women in these industries [2]. It has been suggested that the higher rates of sickness absence due to mental disorders in female-dominated industries may be partly caused by a deteriorating psychosocial work environment in these settings [3]. However, to date, there is a lack of knowledge regarding whether the psychosocial work environment has developed differently in industries based on their gender composition at industry level in Sweden. The rational for the present study is to contribute with knowledge applicable to the design of healthy workplaces and actions taken to prevent occupational risk factors. Working life is organized in industries and workplaces, not by occupation nor gender. This means that work life research that facilitates implementation of the findings, should follow the logic of the agents and arenas where the responsibility over the work environment is placed, such as employers, job designers, human resources departments and production engineers in organizations and establishments. Furthermore, it was concluded in a recent report by the Swedish Social Insurance Agency that the industry level is relevant in explaining some of the variation in sick leave rates on the Swedish labour market [4].

The labour market around the world is highly gender segregated [5] with women and men found in different occupations and industries (horizontal segregation) and women in positions with less power and lower status than men (vertical segregation) [6, 7]. In Sweden, the welfare sector, employing mostly women, was expanded in the 1970’s and Sweden was, for a long time, one of the most horizontally gender segregated countries in Europe [8]. Currently, although gender segregation in Sweden is more alike that of other European countries, men and women are still to a large extent employed in different occupations and industries [3, 9].

Health differences associated with the gender segregation in the labour market may be a reflection of how work is organized, valued and practiced depending on whether it is male or female gendered [6]. However, working life is gendered not only with regards to gender composition in industries, workplaces and occupations; according to gender theory, different job tasks are assigned gendered symbolic meanings [6] and status [10]. For example, technical work such as working with ‘things’ and ‘data’ [11] has been associated with high skill requirements and masculine connotations, such as rationality [12]. On the other hand, caring work, which according to Kohn & Schooler’s classification can be defined as working with ‘humans’ [11], has been given feminine connotations, including that the required skills are by nature embodied in women [13]. If the differently gendered segments of the labour market develop in different directions with regards to psychosocial working conditions, this may over time lead to increased gender differences in work-related health [14].

In the 1990s, the public sector in the Nordic countries went through large reorganizations and cuts and new public management was introduced (NPM) [15, 16]. NPM was a wave of public sector reforms aimed at creating a more efficient and market-driven public sector [17]. In Sweden, large industries organized by the public sector are, for example, the female-dominated industries health care and education. Studies investigating the consequences of the 1990’s economic situation and the introduction of NPM on the psychosocial work environment in the public sector in Sweden show that the organizational changes resulted in a deterioration of the work environment and professional autonomy of the employees [18–20].

The increased risk of mental ill-health in female-dominated industries that has been observed during the past years in Sweden may thus be due to a continuous deterioration of the psychosocial work environment in these industries compared with gender-mixed and male-dominated industries [21–23].

The dimensions of the demand-control-support model [24] have been identified as relevant to study these organizational changes [25], and it is well established that high job demands, low job control and poor social support [24] are associated with mental ill-health and sickness absence [26–29]. Variance in job demands and control have mainly been explored by comparing occupations and sex, but few studies have shown that variance can be attributed to organizational [30, 31] and workplace levels [32].

To the best of our knowledge there is no study focusing on the development and distribution of high job demands, low decision authority and poor social support based on gender composition at the industry level in Sweden. The present study aims to, with an exploratory focus, address this limitation.

### Aim

This study aims to explore the development of job demands, decision authority and social support within and between industries with different gender composition in Sweden. The specific research questions are whether high job demands, low decision authority and poor social support among women and men: i) have changed over the study period within the industries; ii) differ between industries over the study period.

## Methods

### Study population

The respondents are employees living in Sweden, aged 16–64 years, who were not absent, due to sickness or other types of leave, for at least 3 months prior to the data collection. Data were derived from the Swedish Work Environment Survey (SWES) conducted biennially since 1989 by the Swedish Work Environment Authority in collaboration with Statistics Sweden. SWES participants are sampled from the biennial Labour Force Survey (LFS). More than 20,000 persons are randomly drawn from the whole Swedish population, and are stratified by county, sex, citizenship and employment status. Further on, a random sub-sample of gainfully employed people from LFS are sent self-completion SWES questionnaires [33]. These questionnaires contain, among other, questions about the psychosocial work environment.

The current study uses cross-sectional data from 12 waves of SWES (1991 to 2013) and comprises in total 109,698 respondents. The response rate has decreased from 87.2% in 1991 to 58.7% in 2013, with a mean response rate of 75.1% over the 12 waves [3]. The response rate by year of SWES is provided in the additional file 1. Participation across waves has been lower for men and among individuals with low education, low income, and with foreign background. Furthermore, it has also been lower among the self-employed and among individuals with part-time or short-term employment contracts.

SWES data were linked to registry data from the Longitudinal integration database for health insurance and labour market studies (LISA) which annually integrates data on the labour market, educational and social sectors on all individuals that are more than 16 years of age registered in Sweden. In the current study, the labour market industry that the respondent was employed in at the time of the data collections, as well as the participants’ highest degree of education, were drawn from LISA.

### Study variables

#### Industry classification

The industry variable is based on the Swedish Standard Industrial Classification (SNI), a system that follows the recommendations of the Statistical classification of economic activities in the European Community (NACE), allowing for comparisons of data at the national and international level, and over time. The SNI is primarily an activity classification, with production units classified according to the main economic activity carried out. The SNI was last revised in 2007. In the present study, the SNI classification system from 2002 is used for the waves 1991 to 2007, and the SNI 2007 classification for the waves 2009 to 2013.

The SNI 2002 and 2007 have 17 and 21 main categories, where 15 and 19 are applicable for classifying paid work, respectively. For the present study, these categories were grouped together into seven new categories according to two principles: 1) the type of work conducted in each industry, and 2) the proportion of men and women employed in each industry (see Table 1). This classification was used as a proxy for female-dominated, male-dominated and gender-mixed industries. Participants were classified into two male-dominated industries (Goods and energy production and Machinery operations), three gender-mixed industries (Labour intensive services, Knowledge intensive services, and Public administration) and two female-dominated industries (Education and Health and social care). In the two male-dominated categories, the core activity is handling “things” whereas in the two female-dominated categories, the core activity is contact with humans. The three gender-mixed categories encompass activities with varying work objects: handling things, data and humans [11].

**Table 1:** Industry classification according to gender composition

Industry	SNI 2002 category and code	SNI 2007 category and code	Proportion of women in SWES (1991–2013)
Education	• Education (M)	• Education (P)	77.30%
Health and social care	• Health and social work (N)	• Human health and social work activities (Q)	86.85%
Labor intensive services	• Wholesale and retail trade, repair of motor vehicles, motorcycles, personal and household goods (G) • Hotels and restaurants (H) • Other community, social and personal service activities (O)	• Wholesale and retail trade, repair of motor vehicles and motorcycles (G) • Accommodation and food service activities (I) • Arts, entertainment and recreation (R) • Other service activities (S) • Administrative and support service activities (N)	52.65%
Knowledge intensive services	• Financial intermediation (J) • Real estate, renting and business activities (K)	• Information and communication (J) • Financial and insurance activities (K) • Real estate activities (L) • Professional, scientific and technical activities (M)	45.93%
Public administration	• Public administration and defence; compulsory social security (L)	• Public administration and defence; compulsory social security (O)	53.81%
Goods and energy production	• Mining and quarrying (C) • Manufacturing (D) • Electricity, gas and water supply (E)	• Mining and quarrying (B) • Manufacturing (C) • Electricity, gas and air conditioning supply (D) • Water supply; sewerage, waste management and remediation activities (E)	26.51%
Machinery operations	• Agriculture, hunting and forestry (A) • Fishing (B) • Construction (F) • Transport, storage and communication (I)	• Agriculture, forestry and fishing (A) • Construction (F) • Transportation and storage (H)	21.03%

The proportion of women in each industry over the study period (1991–2013) in SWES is given in the last column of Table 1.

### Psychosocial work factors

The psychosocial work factors used in this study were drawn from SWES 1991–2013: job demands, decision authority and social support.

The variables job demands and decision authority were based on four items each whereas social support was derived from two separate questions. These variables serve as indicators of the demand-control-support model dimensions [34] and have been used in previous studies [34–36]. According to the above procedure, respondents were classified as having high job demands if they reported at least two of the following: “have to skip lunch, work late or take work home” at least once every week; at least half of the time “does not have time to talk or even think of anything other than work”; “the work requires undivided attention and concentration” nearly all the time; and agree fully or to some extent to “having far too much to do” (Chronbach’s alpha 0.64). Respondents were classified as having low decision authority if they reported two of the following: “possibility to set the work tempo” half of the time or less; mostly not or never “possibility to decide when various tasks are to be done”; never or mostly “not involved in planning the work”; and agree fully or to some extent to “having too little influence at work” (Cronbach’s alpha 0.63). Respondents were classified as having poor social support if they reported that mostly not or never “received support” from the superiors and/or colleagues “when the work becomes troublesome” (Chronbach’s alpha 0.64). The original response items and scales used to compose each of the variables can be found in the additional file 2.

### Covariates

Age and education of the participants were derived from registry data. Age was categorized in five groups: 16–25, 26–35, 36–45, 46–55, 56–64. The highest degree of education of the respondents was classified into five categories: compulsory education, two years of upper secondary school, three or four years of upper secondary school, university or equivalent less than three years, and university or equivalent three years or more.

### Statistical methods

The proportion of respondents reporting to be exposed to the studied psychosocial work factors between 1991 and 2013 in industries with different gender composition were calculated. Due to changes in the SNI classification system over time, the analyses were conducted separately for periods using the same industry classification: 1991 to 2007 and 2009 to 2013. Because of decreasing response rates, the use of weighted data based on sex and occupation, provided by Statistics Sweden, was considered. However, the variation between the proportions was lower than 3% between weighted and unweighted data. As the use of weighted data restricted the statistical analyses that were possible to perform, unweighted data was used. To estimate if the adverse psychosocial work factors varied between the years when comparing to the first year of the study (1991), logistic regressions were conducted, adjusted for age. SWES data were pooled in 5 groups: 1991 (the reference category), 1993 to 1995, 1997 to 2001, 2003 to 2007 and 2009 to 2013. Estimates are reported as Average Marginal Effects, which can be interpreted as the average change in the probability (0 to 1) of reporting exposure to high job demands, low decision authority and poor social support in each of the group years compared with the reference group. The SWES wave of 1991 was used as the reference category, as it is the year before the Swedish economic crisis, when work conditions were expected to be better than in later years [37]. Estimates show the discrete change from this category with 95% confidence interval. To estimate if the psychosocial work factors differed between industries and between the earlier and later periods of the study, SWES data was pooled into four groups. The first group comprises data from the beginning of the study period (1991, 1993 and 1995), the second group and third group uses data from the middle of the study period (1997, 1999 and 2001; 2003, 2005 and 2007, respectively). The fourth group uses data from the end of the study period (2009, 2011 and 2013). Logistic regressions were conducted, adjusted for age, education and year of data collection. Estimates are reported as Average Marginal Effects, which can be interpreted as the average change in the probability (0 to 1) of reporting exposure to high job demands, low decision authority and poor social support in each industry compared with the reference group. Knowledge intensive services was used as the reference category, as it is a gender-mixed industry and employs highly skilled workers in the private sector, often associated with good psychosocial work conditions. Estimates show the discrete change from this category with 95% confidence interval. All analyses were stratified by sex and performed in STATA version 15.1 for Mac™. The study is reported in adherence with the STROBE guidelines.

## Results

As shown in Table 2, the highest proportion of male respondents were employed in the goods and energy production (28.9%) and the lowest number in health and social care (4.9%). Among women, the highest number of respondents were employed in health and social care (30%), and the lowest in machinery operations (5.6%).

**Table 2:** Characteristics of the study participants in the whole study sample (1991–2013)

	Men *N* = 53,118		Women *N* = 56,580	
Industry				
Goods and energy production	14,958	28.87%	5395	9.74%
Machinery operations	11,571	22.33%	3082	5.56%
Labour intensive services	9337	18.02%	10,382	18.74%
Knowledge intensive services	7200	13.89%	6116	11.04%
Public administration	3315	6.40%	3862	6.97%
Education	2925	5.64%	9960	17.98%
Health and Social care	2513	4.85%	16,592	29.96%
Age Groups				
16–25	4825	9.08%	5525	9.77%
26–35	11,691	22.01%	11,408	20.17%
36–45	13,607	25.62%	14,774	26.12%
46–55	14,110	26.57%	15,538	27.47%
56–65	8879	16.72%	9327	16.49%
Education				
Compulsory	11,292	21.29%	12,703	22.47%
2 years upper secondary	15,275	28.79%	13,381	23.67%
3 or 4 year upper secondary	14,014	26.42%	11,622	20.56%
University less than 3 years	4779	9.01%	7793	13.79%
University, 3 years or more	7690	14.50%	11,028	19.51%

The educational level differed between genders and industries (results available in additional file 1). For example, in education, 50.7% of the men and 38.7% of the women had at least 3 years of university education. In health and social care, 37.8% of the men and 18.6% of the women had 3 years of university education or more.

**Fig. 1:**
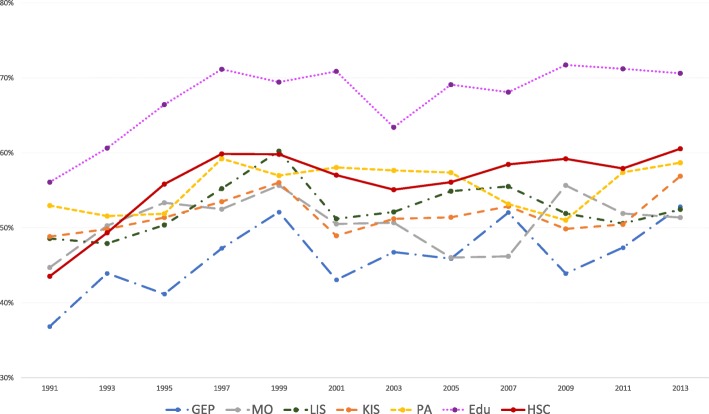
Proportion of women with high job demands, per industry, over the study period Y axis: Proportion of women with high job demands GEP: Goods and energy production; MO: Machinery operations; LIS: Labour intensive services; KIS: Knowledge intensive services; PA: Public administration; Edu: Education; HSC: Health and social care

**Fig. 2:**
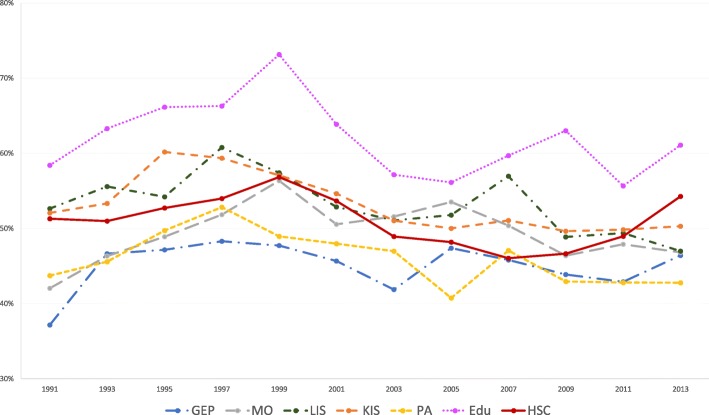
Proportion of men with high job demands, per industry, over the study period Y axis: Proportion of men with high job demands GEP: Goods and energy production; MO: Machinery operations; LIS: Labour intensive services; KIS: Knowledge intensive services; PA: Public administration; Edu: Education; HSC: Health and social care

**Fig. 3:**
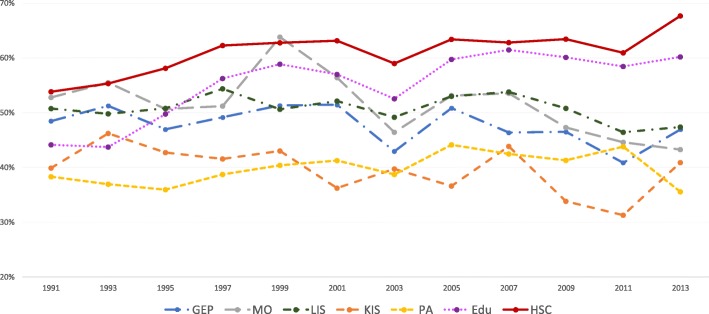
Proportion of women with low decision authority, per industry, over the study period Y axis: Proportion of women with low decision authority GEP: Goods and energy production; MO: Machinery operations; LIS: Labour intensive services; KIS: Knowledge intensive services; PA: Public administration; Edu: Education; HSC: Health and social care

**Fig. 4:**
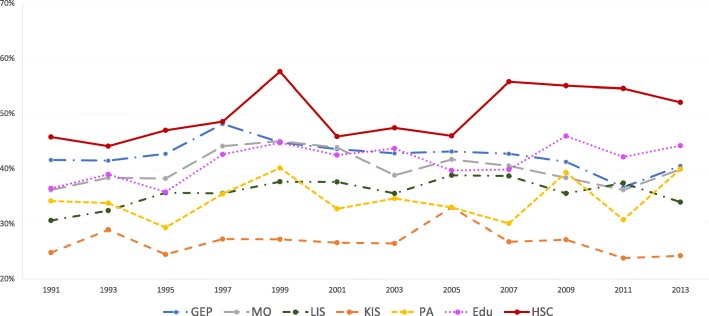
Proportion of men with low decision authority, per industry, over the study period Y axis: Proportion of men with low decision authority GEP: Goods and energy production; MO: Machinery operations; LIS: Labour intensive services; KIS: Knowledge intensive services; PA: Public administration; Edu: Education; HSC: Health and social care

**Fig. 5:**
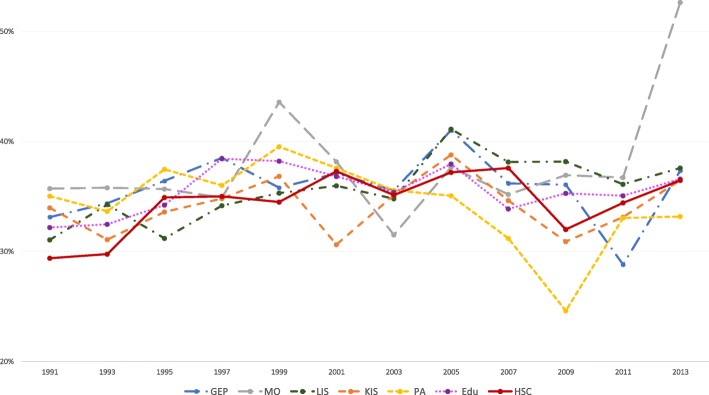
Proportion of women with poor social support, per industry, over the study period Y axis: Proportion of women with poor social support GEP: Goods and energy production; MO: Machinery operations; LIS: Labour intensive services; KIS: Knowledge intensive services; PA: Public administration; Edu: Education; HSC: Health and social care

**Fig. 6:**
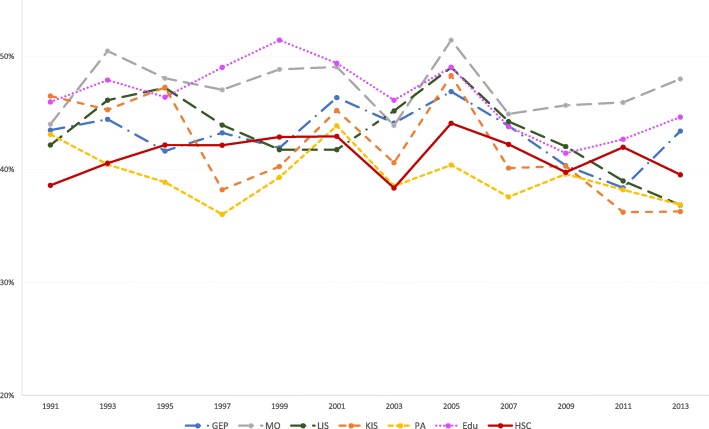
Proportion of men with poor social support, per industry, over the study period Y axis: Proportion of men with poor social support GEP: Goods and energy production; MO: Machinery operations; LIS: Labour intensive services; KIS: Knowledge intensive services; PA: Public administration; Edu: Education; HSC: Health and social care

The results of the statistical analyses will be described in the sub-sections below. The proportions of women and men with high job demands (Figs. 1 and 2), low decision authority (Figs. 3 and 4) and poor social support (Figs. 5 and 6), over time and per industry are shown in the figures. The corresponding proportions are given in the table on the additional files 3 and 4.

### Female-dominated industries

#### Education

Women in education had the highest proportions of high job demands over the study period. When comparing with 1991, the probabilities of having high job demands were higher in all other time periods (see Table 3). In comparison with women in knowledge intensive services, women in education had increased probabilities of having high job demands, over the study period (see Table 4). The probability is higher in the later wave group, between 2009 and 2011 (AME 0.17, 95% CI 0.13, 0.20).

**Table 3:** AMEs for each psychosocial work factor over each time period, stratified by sex

	High job demands				
	Men			Women	
	AME	95% CI		AME	95% CI
Education			Education		
1991	0.00		1991	0.00	
93-95	0.06	0.00 ; 0.12	93-95	**0.06**	0.03 ; 0.09
97-99-01	**0.09**	0.03 ; 0.15	97-99-01	**0.13**	0.10 ; 0.15
03-05-07	-0.02	-0.08 ; 0.05	03-05-07	**0.09**	0.06 ; 0.12
09-11-13	0.00	-0.06 ; 0.07	09-11-13	**0.12**	0.09 ; 0.15
Health and social care			Health and social care		
1991	0.00		1991	0.00	
93-95	0.00	-0.07 ; 0.07	93-95	**0.08**	0.06 ; 0.11
97-99-01	0.03	-0.05 ; 0.10	97-99-01	**0.14**	0.12 ; 0.17
03-05-07	-0.05	-0.12 ; 0.02	03-05-07	**0.12**	0.09 ; 0.14
09-11-13	-0.03	-0.11 ; 0.04	09-11-13	**0.14**	0.11 ; 0.17
Labour intensive services			Labour intensive services		
1991	0.00		1991	0.00	
93-95	0.02	-0.02 ; 0.06	93-95	0.01	-0.03 ; 0.04
97-99-01	**0.05**	0.01 ; 0.08	97-99-01	**0.07**	0.03 ; 0.10
03-05-07	0.00	-0.04 ; 0.04	03-05-07	**0.05**	0.02 ; 0.09
09-11-13	-0.04	-0.08 ; 0.00	09-11-13	0.03	-0.01 ; 0.06
Knowledge intensive services			Knowledge intensive services		
1991	0.00		1991	0.00	
93-95	**0.04**	0.00 ; 0.09	93-95	0.02	-0.04 ; 0.07
97-99-01	**0.05**	0.01 ; 0.09	97-99-01	0.04	-0.01 ; 0.09
03-05-07	-0.01	-0.06 ; 0.03	03-05-07	0.03	-0.02 ; 0.07
09-11-13	-0.02	-0.07 ; 0.02	09-11-13	0.03	-0.03 ; 0.08
Public administration			Public administration		
1991	0.00		1991	0.00	
93-95	0.02	-0.02 ; 0.06	93-95	-0.02	-0.08 ; 0.05
97-99-01	**-0.05**	-0.09 ; -0.01	97-99-01	0.05	-0.01 ; 0.10
03-05-07	**0.04**	0.02 ; 0.07	03-05-07	0.03	-0.03 ; 0.09
09-11-13	**-0.11**	; -0.07	09-11-13	0.02	-0.04 ; 0.08
Goods and energy production			Goods and energy production		
1991	0.00		1991	0.00	
93-95	**0.10**	0.07 ; 0.13	93-95	**0.06**	0.01 ; 0.11
97-99-01	**0.10**	0.08 ; 0.13	97-99-01	**0.11**	0.06 ; 0.15
03-05-07	**0.08**	0.05 ; 0.11	03-05-07	**0.11**	0.06 ; 0.16
09-11-13	**0.07**	0.04 ; 0.10	09-11-13	**0.10**	0.05 ; 0.15
Machinery operations			Machinery operations		
1991	0.00		1991	0.00	
93-95	**0.05**	0.02 ; 0.09	93-95	**0.07**	0.01 ; 0.13
97-99-01	**0.11**	0.08 ; 0.14	97-99-01	**0.08**	0.02 ; 0.14
03-05-07	**0.10**	0.07 ; 0.13	03-05-07	0.03	-0.03 ; 0.09
09-11-13	**0.05**	0.01 ; 0.08	09-11-13	**0.08**	0.01 ; 0.15
Low decision authority					
	Men			Women	
	Model 1			Model 1	
	AME	95% CI		AME	95% CI
Education			Education		
1991	0.00		1991	0.00	
93-95	0.01	-0.06 ; 0.08	93-95	0.02	-0.01 ; 0.06
97-99-01	**0.07**	0.00 ; 0.14	97-99-01	**0.13**	0.09 ; 0.16
03-05-07	0.05	-0.02 ; 0.11	03-05-07	**0.13**	0.10 ; 0.16
09-11-13	**0.08**	0.01 ; 0.15	09-11-13	**0.14**	0.11 ; 0.18
Health and social care			Health and social care		
1991	0.00		1991	0.00	
93-95	0.01	-0.06 ; 0.08	93-95	**0.03**	0.00 ; 0.06
97-99-01	0.07	0.00 ; 0.14	97-99-01	**0.09**	0.07 ; 0.12
03-05-07	**0.07**	0.00 ; 0.14	03-05-07	**0.09**	0.06 ; 0.11
09-11-13	**0.12**	0.05 ; 0.19	09-11-13	**0.10**	0.08 ; 0.13
Labour intensive services			Labour intensive services		
1991	0.00		1991	0.00	
93-95	**0.04**	0.00 ; 0.08	93-95	0.00	-0.04 ; 0.03
97-99-01	**0.08**	0.04 ; 0.11	97-99-01	0.02	-0.01 ; 0.06
03-05-07	**0.10**	0.06 ; 0.13	03-05-07	0.02	-0.02 ; 0.05
09-11-13	**0.08**	0.04 ; 0.12	09-11-13	-0.01	-0.05 ; 0.02
Knowledge intensive services			Knowledge intensive service		
1991	0.00		1991	0.00	
93-95	0.02	-0.02 ; 0.07	93-95	0.05	0.00 ; 0.10
97-99-01	0.02	-0.02 ; 0.07	97-99-01	0.00	-0.04 ; 0.05
03-05-07	**0.05**	0.01 ; 0.09	03-05-07	0.00	-0.04 ; 0.05
09-11-13	0.02	-0.03 ; 0.06	09-11-13	-0.05	-0.10 ; 0.00
Public administration			Public administration		
1991	0.00		1991	0.00	
93-95	-0.02	-0.08 ; 0.04	93-95	-0.01	-0.08 ; 0.05
97-99-01	0.03	-0.03 ; 0.09	97-99-01	0.02	-0.04 ; 0.08
03-05-07	0.01	-0.06 ; 0.07	03-05-07	0.04	-0.02 ; 0.10
09-11-13	0.03	-0.03 ; 0.09	09-11-13	0.03	-0.03 ; 0.10
Good and energy production			Good and energy production		
1991	0.00		1991	0.00	
93-95	0.01	-0.02 ; 0.04	93-95	0.01	-0.04 ; 0.06
97-99-01	**0.05**	0.02 ; 0.08	97-99-01	0.02	-0.02 ; 0.07
03-05-07	**0.03**	0.00 ; 0.06	03-05-07	-0.01	-0.06 ; 0.04
09-11-13	0.00	-0.03 ; 0.03	09-11-13	-0.03	-0.09 ; 0.02
Machinery operations			Machinery operations		
1991	0.00		1991	0.00	
93-95	0.03	-0.01 ; 0.06	93-95	0.00	-0.06 ; 0.06
97-99-01	**0.09**	0.06 ; 0.12	97-99-01	0.04	-0.01 ; 0.10
03-05-07	**0.05**	0.02 ; 0.09	03-05-07	-0.01	-0.07 ; 0.05
09-11-13	0.03	0.00 ; 0.07	09-11-13	-0.07	-0.14 ; 0.00
Poor social support					
	Men			Women	
	Model 1			Model 1	
	AME	95% CI		AME	95% CI
Education			Education		
1991	0.00		1991	0.00	
93-95	0.01	-0.06 ; 0.08	93-95	0.01	-0.03 ; 0.05
97-99-01	0.03	-0.03 ; 0.10	97-99-01	**0.05**	0.01 ; 0.09
03-05-07	-0.01	-0.08 ; 0.06	03-05-07	0.03	-0.01 ; 0.07
09-11-13	-0.05	-0.12 ; 0.02	09-11-13	0.02	-0.02 ; 0.06
Health and social care			Health and social care		
1991	0.00		1991	0.00	
93-95	0.02	-0.05 ; 0.10	93-95	**2.00**	0.00 ; 0.06
97-99-01	0.03	-0.04 ; 0.10	97-99-01	**0.06**	0.04 ; 0.09
03-05-07	0.01	-0.06 ; 0.08	03-05-07	**0.07**	0.04 ; 0.10
09-11-13	0.00	-0.07 ; 0.08	09-11-13	**0.05**	0.02 ; 0.08
Labour intensive services			Labour intensive services		
1991	0.00		1991	0.00	
93-95	**0.04**	0.00 ; 0.08	93-95	0.02	-0.02 ; 0.05
97-99-01	-0.01	-0.04 ; 0.03	97-99-01	**0.04**	0.00 ; 0.08
03-05-07	**0.02**	-0.01 ; 0.06	03-05-07	**0.07**	0.03 ; 0.11
09-11-13	-0.05	-0.09 ; -0.01	09-11-13	**0.06**	0.02 ; 0.09
Knowledge intensive services			Knowledge intensive services		
1991	0.00		1991	0.00	
93-95	-0.01	-0.05 ; 0.04	93-95	-0.02	-0.07 ; 0.03
97-99-01	**-0.05**	-0.10 ; -0.01	97-99-01	0.00	-0.04 ; 0.05
03-05-07	-0.04	-0.08 ;	03-05-07	0.02	-0.03 ; 0.06
09-11-13	**-0.11**	-0.15 ; -0.06	09-11-13	-0.01	-0.06 ; 0.04
Public administration			Public administration		
1991	0.00		1991	0.00	
93-95	-0.04	-0.10 ; 0.02	93-95	-0.01	-0.07 ; 0.06
97-99-01	-0.05	-0.11 ; 0.01	97-99-01	0.01	-0.05 ; 0.07
03-05-07	**-0.06**	-0.12 ; 0.00	03-05-07	-0.03	-0.08 ; 0.03
09-11-13	**-0.06**	-0.12 ; 0.00	09-11-13	-0.06	-0.12 ; 0.00
Good and energy production			Good and energy production		
1991	0.00		1991	0.00	
93-95	-0.01	-0.04 ; 0.02	93-95	0.02	-0.03 ; 0.07
97-99-01	0.00	-0.03 ; 0.03	97-99-01	0.04	-0.01 ; 0.09
03-05-07	0.01	-0.02 ; 0.04	03-05-07	0.04	-0.01 ; 0.09
09-11-13	-0.04	-0.07 ; -0.01	09-11-13	0.00	-0.06 ; 0.05
Machinery operations			Machinery operations		
1991	0.00		1991	0.00	
93-95	**0.04**	0.01 ; 0.08	93-95	0.00	-0.06 ; 0.06
97-99-01	**0.03**	0.00 ; 0.06	97-99-01	0.02	-0.03 ; 0.08
03-05-07	0.01	-0.02 ; 0.05	03-05-07	-0.01	-0.07 ; 0.05
09-11-13	0.00	-0.04 ; 0.03	09-11-13	0.04	-0.03 ; 0.11

Entries in bold where the lower value is 0.00 it has been confirmed that is significant (p value). Interval does not contain 0 as it has been confirmed with the milesimal numbers

**Table 4:** AME and 95% CI for adverse work factors in the different industries for women

Women								
	1991 - 1993 - 1995		1997 - 1999 - 2001		2003 - 2005 - 2007		2009 - 2011 - 2013	
	AME	95% CI	AME	95% CI	AME	95% CI	AME	95% CI
High demands								
Knowledge intensive services	0.00		0.00		0.00		0.00	
Education	**0.05**	0.02 ; 0.08	**0.13**	0.10 ; 0.16	**0.12**	0.09 ; 0.15	**0.17**	0.13 ; 0.20
Health and social care	0.00	-0.03 ; 0.03	**0.06**	0.03 ; 0.09	**0.04**	0.01 ; 0.07	**0.07**	0.04 ; 0.11
Labour intensive services	0.01	-0.01 ; 0.04	**0.05**	0.02 ; 0.08	**0.04**	0.01 ; 0.07	0.03	-0.01 ; 0.06
Public administration	0.00	-0.03 ; 0.04	0.03	-0.01 ; 0.07	0.03	-0.01 ; 0.06	0.02	-0.02 ; 0.07
Goods and energy production	**-0.06**	-0.10 ; -0.03	-0.02	-0.05 ; 0.02	-0.02	-0.06 ; 0.02	-0.02	-0.07 ; 0.02
Machinery operations	0.01	-0.02 ; 0.05	0.02	-0.02 ; 0.06	-0.02	-0.07 ; 0.02	0.04	-0.01 ; 0.10
Low decision authority								
Knowledge intensive services	0.00	;	0.00		0.00		0.00	
Education	**0.05**	0.02 ; 0.08	**0.19**	0.16 ; 0.21	**0.20**	0.17 ; 0.22	**0.25**	0.20 ; 0.28
Health and social care	**0.13**	0.10 ; 0.15	**0.22**	0.19 ; 0.24	**0.22**	0.19 ; 0.24	**0.27**	0.24 ; 0.30
Labour intensive services	**0.06**	0.03 ; 0.09	**0.10**	0.07 ; 0.13	**0.09**	0.06 ; 0.12	**0.11**	0.07 ; 0.14
Public administration	**-0.05**	-0.09 ; -0.01	0.02	-0.02 ; 0.05	**0.04**	0.00 ; 0.08	**0.07**	0.03 ; 0.12
Goods and energy production	**0.05**	0.01 ; 0.08	**0.09**	0.05 ; 0.12	**0.05**	0.02 ; 0.09	**0.08**	0.03 ; 0.12
Machinery operations	**0.09**	0.06 ; 0.13	**0.14**	0.11 ; 0.18	**0.09**	0.05 ; 0.13	**0.08**	0.02 ; 0.13
Lack of support								
Knowledge intensive services	0.00		0.00		0.00		0.00	
Education	-0.03	-0.06 ; 0.00	0.02	-0.01 ; 0.05	-0.02	-0.05 ; 0.01	0.01	-0.03 ; 0.04
Health and social care	-0.01	-0.04 ; 0.01	0.01	-0.02 ; 0.04	0.00	-0.03 ; 0.02	0.00	-0.03 ; 0.04
Labour intensive services	0.01	-0.02 ; 0.04	0.02	-0.01 ; 0.05	0.03	0.00 ; 0.06	**0.05**	0.02 ; 0.09
Public administration	0.02	-0.02 ; 0.05	0.02	-0.02 ; 0.06	**-0.03**	-0.07 ; 0.00	-0.04	-0.08 ; 0.00
Goods and energy production	**0.03**	0.00 ; 0.07	**0.04**	0.00 ; 0.07	0.02	-0.02 ; 0.05	0.00	-0.04 ; 0.05
Machinery operations	**0.04**	0.00 ; 0.08	**0.05**	0.01 ; 0.09	-0.01	-0.05 ; 0.04	**0.08**	0.02 ; 0.14

* Adjusted for age group, highest degree of education and year of SWES

Entries in bold where the lower value is 0.00 it has been confirmed that is significant (p value). Interval does not contain 0 as it has been confirmed with the milesimal numbers

Women in this industry also reported that decision authority decreased over the study period. Comparing with 1991, the probabilities of having low decision authority varied between 13 to 14%, from 1997 to 2013. In comparison with women in knowledge intensive services, the probabilities have been increasing over the four study group periods, with the highest probability in the later studied group period (AME 0.25, 95% CI 0.20, 0.28).

In 1997, 38% of women reported having poor social support at work. Between 1997 and 2001, there was a 5% higher probability of reporting low social support when comparing to 1991.

Men in education reported the highest proportions of having high job demands, from all the study participants. Increased probabilities of having high job demands were observed between 1997 and 2001 (AME 0.09, CI 95% 0.06, 0.12), when comparing to 1991. However, when comparing to men in knowledge intensive services, the probabilities of having high job demands varied between 18 and 24%, the highest observed between 2009 and 2011 (AME 0.24, 95% CI 0.19, 0.28) (see Table 5).

**Table 5:** AME and 95% CI for adverse work factors in the different industries for men

Men								
	1991 - 1993 - 1995		1997 - 1999 - 2001		2003 - 2005 - 2007		2009 - 2011 - 2013	
	AME	95% CI	AME	95% CI	AME	95% CI	AME	95% CI
High demands								
Knowledge intensive services	0.00		0.00		0.00		0.00	
Education	0.02	-0.02 ; 0.06	**0.08**	0.04 ; 0.12	**0.05**	0.00 ; 0.09	**0.08**	0.03 ; 0.13
Health and social care	**-0.05**	-0.09 ; -0.01	-0.03	-0.08 ; 0.02	-0.04	-0.08 ; 0.01	0.00	-0.05 ; 0.05
Labour intensive services	**0.04**	0.02 ; 0.07	0.03	0.00 ; 0.06	**0.05**	0.01 ; 0.08	0.01	-0.03 ; 0.05
Public administration	**-0.11**	-0.14 ; -0.07	**-0.08**	-0.12 ; -0.04	**-0.07**	-0.11 ; -0.02	**-0.08**	-0.12 ; -0.03
Goods and energy production	**-0.06**	-0.08 ; -0.03	**-0.07**	-0.09 ; -0.04	**-0.03**	-0.06 ; -0.01	-0.03	-0.07 ; 0.00
Machinery operations	-0.02	-0.05 ; 0.00	0.00	-0.03 ; 0.02	**0.04**	0.01 ; 0.07	0.01	-0.03 ; 0.04
Low decision authority								
Knowledge intensive services	0.00		0.00		0.00		0.00	
Education	**0.18**	0.13 ; 0.22	**0.22**	0.18 ; 0.26	**0.18**	0.14 ; 0.22	**0.24**	0.19 ; 0.28
Health and social care	**0.21**	0.17 ; 0.26	**0.26**	0.22 ; 0.30	**0.23**	0.19 ; 0.28	**0.31**	0.26 ; 0.36
Labour intensive services	**0.03**	0.00 ; 0.06	**0.07**	0.04 ; 0.10	**0.06**	0.03 ; 0.09	**0.09**	0.05 ; 0.13
Public administration	**0.10**	0.06 ; 0.14	**0.13**	0.09 ; 0.17	**0.07**	0.03 ; 0.12	**0.14**	0.09 ; 0.19
Goods and energy production	**0.13**	0.10 ; 0.15	**0.16**	0.13 ; 0.19	**0.12**	0.09 ; 0.15	**0.13**	0.10 ; 0.17
Machinery operations	**0.08**	0.05 ; 0.11	**0.14**	0.11 ; 0.17	**0.08**	0.05 ; 0.11	**0.11**	0.07 ; 0.15
Lack of support								
Knowledge intensive services	0.00		0.00		0.00		0.00	
Education	-0.01	-0.05 ; 0.03	**0.08**	0.04 ; 0.12	0.01	-0.03 ; 0.06	0.04	-0.01 ; 0.09
Health and social care	**-0.06**	-0.10 ; -0.02	0.01	-0.04 ; 0.06	-0.03	-0.08 ; 0.01	0.02	-0.02 ; 0.08
Labour intensive services	0.00	-0.03 ; 0.03	0.01	-0.02 ; 0.04	0.03	0.00 ; 0.06	0.02	-0.01 ; 0.06
Public administration	**-0.07**	-0.10 ; -0.03	-0.04	-0.07 ; 0.00	**-0.06**	-0.10 ; -0.02	0.00	-0.05 ; 0.05
Goods and energy production	-0.02	-0.05 ; 0.01	0.02	-0.01 ; 0.04	0.01	-0.02 ; 0.04	0.02	-0.01 ; 0.05
Machinery operations	0.02	-0.01 ; 0.05	**0.06**	0.03 ; 0.09	0.03	0.00 ; 0.06	**0.08**	0.04 ; 0.11

* Adjusted for age group, highest degree of education and year of SWES

Entries in bold where the lower value is 0.00 it has been confirmed that is significant (p value). Interval does not contain 0 as it has been confirmed with the milesimal numbers

#### Health and social care

For women in this industry, the probabilities of having high job demands were higher in all study group periods when comparing with 1991. Between 1993 and 1995, the probability was 8% (95% CI 0.03, 0.09), between 1997 and 2001 the probability was 14% (95% CI 0.12, 0.17), between 2003 and 2007 the probability was 12% (95% CI 0.09, 0.14) and in the later study group period it was 14% (95% CI 0.11, 0.17), compared with the reference category. When comparing with women in knowledge intensive services, women in health and social care services had higher probabilities of high job demands from 1997 onwards. In comparison with 1991, the probabilities of having lower decision authority for women in this industry were higher in all later time periods. When comparing with knowledge intensive services, the probabilities varied from 13% in the beginning of the study period (AME 91-93-95 0.13; 95% CI 0.10, 0.15) to 27% in the later part of the study period (AME 09–11-13 0.27, 95% CI 0.24, 0.30). Among women, the probabilities of having poor social support were higher in all other study periods, when comparing to 1991. For men in health and social care, there was 9% increased probability of having high demands between 1997 and 2001 when comparing to 1991 (AME 0.09, 95% CI 0.03, 0.15).

In comparison with men in knowledge intensive services, there was a 5% (95% CI -0.09, − 0.01) lower probability of having high job demands in the beginning of the study period (1991–1995), a value that was non-significant in the following years of the study period. Men in health and social care had increased probabilities of having high demands over the study period when comparing with men in knowledge intensive services, with the highest values in the end of the study period (AME 0.31, 95% CI 0.26, 0.36).

### Gender-mixed industries

#### Labour intensive services

Women in labour intensive services had increased probabilities of having high job demands between 1997 and 2001 and between 2003 and 2007 when comparing with 1991. These results were also significant in these years when comparing the probabilities of having high job demands with women in knowledge intensive services. In comparison with women in knowledge intensive services, women in labour intensive services had higher probabilities of having low decision authority over the study period, with the highest value at the end of the study period (AME 09–11-13 0.11, 95% CI 0.07, 0.14). A similar pattern is verified for men in this industry, in comparison with men in knowledge intensive services, with a higher value at the end of the study period (AME 09-11-13 0.09, 95% CI 0.05, 0.13). Over time, men in this industry had higher probabilities of having lower decision authority in comparison with 1991. In the later years (from 1997 to 2013), women reported higher probabilities of having poor social support in comparison with 1991. Comparing with women in knowledge intensive services, results were significant in the latest years of the study period (AME 09–11-13 0.05, 95% CI 0.02, 0.09).

#### Knowledge intensive services

Men in knowledge intensive services had higher probabilities of having high job demands between 1992 to 1995 and between 1997 to 2001, when comparing with 1991. For low decision authority, the results show 5% higher probability of having this adverse psychosocial work factor in the period 2003 to 2007, when comparing with 1991. When comparing with all other industries except for public administration, men and women working in knowledge intensive services report better psychosocial working conditions. For social support, between 1991 and 2001 and between 2009 to 2013, men in this industry had lower probabilities of having poorer social support, when comparing with 1991.

#### Public administration

Men in public administration had lower probabilities of having high job demands between 1997 and 2001 and between 2009 and 2013. However, between 2003 and 2007, the probabilities were higher when comparing with 1991 (AME 0.04, 95% CI 0.02; 0.07). Comparing with knowledge intensive services, the probabilities have been constantly lower over the study period. Among women, the probabilities of having low decision authority in the beginning of the study period were lower, but from 2003 to 2013 the probabilities have been higher, when comparing with women in knowledge intensive services. For men, the probabilities of having low decision authority in this industry have increased, when comparing to men in knowledge intensive services. Between 2003 and 2007, women in this industry had lower probabilities of having poor social support in comparison with our reference group. For men, the probabilities were also lower in this study period and between 1991 to 1995 of having poor social support, comparing with men in knowledge intensive services.

### Male-dominated industries

#### Goods and energy production

Both men and women in this industry had higher probabilities of high job demands across the study period when comparing with 1991. For men, the probabilities were lower from 1991 to 2007 when comparing to men in knowledge intensive services. For women, increased probabilities of having high job demands were observed between 1991 to 2001, when comparing to women in knowledge intensive services. Both men and women had higher probabilities of low decision authority during the study period when comparing to men and women in knowledge intensive services. Women in goods and energy production also had increased probabilities of poor social support between 1991 and 2001, when comparing to women in knowledge intensive services.

#### Machinery operations

Among women in machinery operations, there were increased probabilities of having high job demands between 1993 and 2001 and from 2009 to 2011, comparing with 1991. For men, the probabilities were increased during the whole study period when comparing with 1991. When comparing with knowledge intensive services, women had decreased probabilities of high job demands (AME -0.06; 95% CI -0.10, − 0.03). For men, the probabilities were decreased between 1991 and 2007, with 7% lower probabilities of having high job demands between 1997 and 2001 (AME -0.07, 95% CI -0.09, − 0.04).

Among men in machinery operation, probabilities of having low decision authority were higher between 1997 and 2007 when comparing with 1991. In comparison with men and women in knowledge intensive services, men and women in machinery operations had increased probabilities of low decision authority during the whole study period, with the highest value for both genders between 1997 and 2001 (AME for women 0.14, 95% CI 0.11, 0.18; AME for men 0.14, 95% CI 0.11, 0.17).

The probabilities of having poor social support were increased for men in machinery operation between 1993 and 1995 and between 1997 and 2001, in comparison with 1991. When comparing with workers in knowledge intensive services, men had increased probabilities of poor social support between 1997 and 2001 and between 2009 and 2013, whereas women had increased probabilities in all studied group periods except between 2003 and 2007.

## Discussion

The current study found that high job demands and low decision authority, and poor social support for women, appear to have become more common in the female-dominated industries education and health and social care over the study period. High job demands were in the later period (2009–2013) more common in both genders in education and among women in health and social care, compared to the reference category. Low decision authority was reported more often in both female-dominated industries over the study period, and the differences compared with the reference category were increased in the later periods, among both women and men. In contrast, poor social support was more common among women in health and social care and in the gender-mixed industry labour intensive services. The results will be discussed below according to industries that are female-dominated, gender-mixed and male-dominated.

### Female-dominated industries

The results show that high job demands and low decision authority are particularly high in education and health and social care. The development of these factors has been worse in these industries, compared to others in the labour market, over the study period. This negative development is particularly pronounced among women.

The increase in the proportions of high job demands and poor decision authority was steeper in the 1990s, for workers in education and health and social care (Figs. 1 and 2). These results could reflect the organizational changes that occurred in these industries, following the economic crisis in the 90s and NPM, which can affect the development of negative health consequences, as suggested in a previous paper [18].

The highest proportions of workers reporting low decision authority were found in health and social care, for both men and women. Thus, poor decision authority appears to be experienced across occupations and genders in this industry, indicating that this is a psychosocial work environment problem to be targeted at the industry level. Low professional autonomy across occupations and positions in health and social care has been found to be a consequence of the introduction of new public management in previous studies [15, 18].

When analysing workers in the female-dominated industries, there are noticeable differences between genders. The proportions of women reporting high job demands have remained on similarly high levels between the end of the 1990s and the later data collections of this study. For men, the high proportions decreased again after the 1990s. Also, for low decision authority, the proportions reported by women have increased further in the later years of the study period, for those in female-dominated industries. The different patterns for women and men may be explained by the fact that, within female-dominated industries, men and women have different occupations (horizontal gender segregation within the industry) and occupy different positions (vertical gender segregation within the industry). Most men in this industry have at least 3 years of university education (see Additional file 1), whereas the majority of women are found in the lowest educational groups, in caring jobs with close contact with patients and clients. A deterioration of job demands and decision authority in caring jobs have previously been reported [18, 38].

The increased risks for mental ill-health in female-dominated industries observed in Sweden in the last years have been hypothesized to be due to poorer psychosocial work environment in these industries compared to others [21–23]. Though no mental ill-health measures have been included in this study, poorer development of job demands and job control for both men and women in these industries has been observed.

### Gender-mixed industries

The proportions of high job demands fluctuated between 1991 and 2007 among both genders in labour intensive services, and among men in knowledge intensive services. The highest proportions were observed in the second part of the 1990s, and the values were similar at the start and end of the study period. This finding is in line with previous research, showing increased job intensity in times of economic crisis also in high-skilled jobs [39]. In public administration, the average marginal effects were lower among men in all time periods, when compared with the reference category of knowledge intensive services.

The proportions of workers reporting low decision authority are the lowest for those in knowledge intensive services and public administration. This finding supports results based on data from European Union (EU) countries, showing that job control has increased in the last decades particularly in high-skilled jobs, and especially so in the Nordic countries [39].

### Male-dominated industries

The highest proportions of poor social support were reported by both men and women in machinery operations. This can be due to the nature of work in this industry, as it can have a somewhat individualistic connotation. For women, in recent years, those working in machinery operations report the highest values of poor social support. This can be interpreted as the gender in minority having worse social support than those in majority, in line with the tokenism theory [40], but further studies are needed, including exploring the dramatic increase in 2013 for women in machinery operations.

The primary focus of the present study was not to compare women and men. However, although the chosen strategy of gender stratified analysis does not allow to make actual gender comparisons, some evident patterns emerged. Many more women seemed to have lower decision authority than men in all industries in our study (Figs. 3 and 4). On the other hand, more men than women reported poorer social support (Figs. 5 and 6), with one exception: women in machinery operations in 2013. Thus, in addition to systematic differences between industries, there seems to be structural gender-related differences across the labour market.

We chose to analyse the development of the psychosocial work factors by industry because of its relevance for the organization of work as this level may be particularly suitable for interventions aimed at improving the psychosocial working conditions. However, our results lend support for that all variation in working conditions may not be captured at the industry level. The results regarding gender differences within industries suggest that there are also conditions that are specific to occupation and positions within industries, as discussed in a recent report by the Swedish Social Insurance Agency [4]. However, further exploration of the present data is needed in order to disentangle to what extent differences are due to variation between occupations and positions, to variations between genders, or both.

### Strengths and limitations

To our knowledge, this study is one of the first of its kind exploring the development of the dimensions of the demand-control-support mode over a long period of time and between industries, considering their gender composition, in a representative sample of the whole Swedish working population.

However, there are several limitations that should be accounted for when interpreting the results. First, the response rate has been decreasing over time, and lower responses rates have been observed for men, individuals with low education, low income and foreign background, across the study period. Hence, it is with caution that these results can be generalized to the Swedish working population as a whole. Second, the data are based on self-reported measures, as most studies on psychosocial working conditions, which are vulnerable to response bias. The measures of the demands, decision authority and support dimensions that were used in the present study are not directly comparable with the standard measures of the demand-control-support model [24]. However, these measures have shown adequate internal consistency and have been used in previous studies, allowing for comparisons between groups and over time [34–36].

The industry classification used in this study considers not only the gender composition but also the type of work of the industry, providing a more scrutinized and complete classification than if only numbers of men and women in each industry were taken into consideration. However, the official categorization of industries changed in 2007. Overall, the classification of industries is very similar for both periods. The two groups that changed between one classification to the other were knowledge intensive services and labour intensive services. This was mainly due to new and growing industries being identified and classified. To overcome this limitation, our analyses of development over time were made in separate time-periods. Although only a small proportion of the populations is classified differently in the two study periods, this should be considered when interpreting our results.

Knowledge intensive services was chosen as the reference category for the analyses of differences between industries. The reason was that knowledge intensive services is a gender-mixed industry employing individuals with high educational levels in high-skilled jobs, often associated with good working conditions. However, high job demands were rather common in this industry over the study period. Larger AMEs of reporting high job demands would have been observed in some industries, if the industry with the best conditions would have been used as the reference category. For interpretability of the results, we decided to use the same reference category for all work dimensions.

In this study, a seven-industry classification was used to explore the development of psychosocial working conditions. In forthcoming studies, this classification could be further explored. For example, as public administration and knowledge intensive services present similar distribution of educational and work exposure levels, they could be merged into one group in future studies. On the other hand, an even more detailed analysis of variation between industries could be explored if using the original 15 or 19 industries in the Swedish Standard Industrial Classification (SNI) system.

It should be noted that all employees at a production unit are classified according to its main activity. This means that the differences between male- and female- dominated industries in working conditions that we have shown, probably are underestimated for occupations in minority. These are administrators, service workers and managers, that are differently “gendered” than the workers performing the core production, and have other job tasks that might affect job demands, control and social support. However, it is noteworthy that in spite of this limitation, several significant differences between industries were found. To get more precise estimates, information on occupation could be used for stratification of workers performing the core tasks from other functions in the production units, for example.

To compare change over time between industries, data was pooled in four time periods, each comprising three waves of SWES. The period between 1991 and 1995 captures not only the early effects of the economic crisis of 1992 [41, 42] but also the early effects of new public management, which affected particularly the public sector, as discussed in previous sections. Using all periods allows for a better understanding of the development of psychosocial working conditions over time.

### Implications for future research and occupational health practice

Given how working life is organized, studying the development of psychosocial work factors over time by industry contributes with applicable knowledge to the design of healthy workplace. This study contributes with an approach that opens future research to link the fields of production technology and work organization with the work environment and occupational health, as previously requested [43–45]. If only gender, class or occupation are compared when exploring changes of work environment over time, it is not possible to disentangle whether changes in work conditions are due to structural changes in industries, effects of work environment improvements or due to changes of the occupational distribution of the labour force [46]. Industries tend to use similar technologies and management strategies. Hence, it is reasonable to believe that larger structural and technological changes in an industry, such as in health and social care during the 1990s, also lead to changes in job tasks and composition of occupations [47]. Future longitudinal research comparing units that are linked to the production process, such as industries, should advance knowledge on the pathways to healthy or hazardous work.

The present study also contributes to the research field of stratification, inequality and gendered processes in working life. The results indicate inertia of horizontal division of labour in industries with different types of operations or work objects. Since a few decades ago, industries with the core operation aimed at “handle material things” employ almost half of the male workforce in Sweden. On the other hand, almost half of the female workforce has “humans” as work objects, organized in two large welfare services. One mechanism to be explored in future research concerns how operations with different work objects are “gendered” and associated with different status, and how this affects the workers influence in periods of structural changes, as indicated in the present study.

## Conclusions

The present study suggests that there has been a negative development of job demands and decision authority in the female-dominated industries, such as education and health and social care, in Sweden. Social support has developed more negatively in the male-dominated industry of machinery operations for both genders, and also for women labour intensive services. As working life is organized by industries and workplaces, this knowledge may contribute to the design of healthier workplaces and prevention of occupational risk factors. The extent to which the negative development in psychosocial work factors can explain the recent increase in mental ill-health and sickness absence for mental disorders in these industries may be investigated in future studies.

## Additional files

Additional file 1: Response rates by year of SWES. (PDF 32 kb)

Additional file 2: Composition of the study outcome variables. (PDF 50 kb)

Additional file 3: Proportion of women exposed to each psychosocial work factor in each wave by industry (PDF 61 kb)

Additional file 4: Proportion of men exposed to each psychosocial work factor in each wave by industry (PDF 61 kb)

## Abbreviations

AME: Average Marginal Effects
CI: Confidence interval
LISA: Longitudinal integration database for health insurance and labour market studies
NACE: Statistical classification of economic activities in the European Community
NPM: New public management
SNI: Swedish Standard Industrial Classification
SWES: Swedish Work Environment Survey
