# Supplementary material for: Development of job demands, decision authority and social support in industries with different gender composition – Sweden, 1991–2013
Source: BMC Public Health. 2019 Jun 14;19:758. doi: 10.1186/s12889-019-6917-8 (PMC6570932; doi:10.1186/s12889-019-6917-8)
Supplement: Supplementary file 1 — Response rates by year of SWES. (PDF 32 kb) [file 12889_2019_6917_MOESM1_ESM.pdf]

Additional file 1: Response rates by year of SWES

Year of SWES	Number of eligible participants	Number of respondents	Response rate
1991	14,300	12,462	87.15%
1993	13,291	11,709	88.10%
1995	13,173	10,830	82.21%
1997	12,798	10,400	81.26%
1999	12,546	9,734	77.59%
2001	12,911	9,542	73.91%
2003	12,372	9,115	73.67%
2005	13,541	9,579	70.74%
2007	10,680	7,615	71.30%
2009	9,159	6,239	68.12%
2011	123,88	7,784	62.84%
2013	8,135	4,689	57.64%
