# Supplementary material for: Development of job demands, decision authority and social support in industries with different gender composition – Sweden, 1991–2013
Source: BMC Public Health. 2019 Jun 14;19:758. doi: 10.1186/s12889-019-6917-8 (PMC6570932; doi:10.1186/s12889-019-6917-8)
Supplement: Supplementary file 2 — Composition of the study outcome variables. (PDF 50 kb) [file 12889_2019_6917_MOESM2_ESM.pdf]

Additional file 2: Composition of the study outcome variables

The composition of each of the outcome variables used in the article are described below, and includes the original response items for each item.

Job demands

- Do you sometimes have so much work to do that you have to skip lunch, work late, or take work home with you?
    - Everyday
    - A couple of days a week (1 of 2 days)
    - One day a week (1 day of 5)
    - A couple of days a month (1 day of 10)
    - Not at all/seldom in the last 3 months

- Is your work sometimes so stressful that you do not have time to talk or even think of anything other than work?
    - Nearly all the time
    - Roughly ¾ of the time
    - Half of the time
    - Roughly ¼ of the time
    - Some (perhaps 1/10 of the time)
    - No, not at all

- Does your work require your undivided attention and concentration?
    - Nearly all the time
    - Roughly ¾ of the time
    - Half of the time
    - Roughly ¼ of the time
    - Some (perhaps 1/10 of the time)
    - No, not at all

- How do you experience your work?
    - Likert scale with 5 response items, going from 1 (far too much to do) to 5 (far too little to do)

Decision authority

- Is it possible for you to set your own work tempo?
    - Nearly all the time
    - Roughly ¾ of the time
    - Half of the time
    - Roughly ¼ of the time
    - Some (perhaps 1/10 of the time)
    - No, not at all

- Is it possible for you to decide on your own when various tasks are to be done (for example, by choosing to work a bit faster some days and taking it easy other days)?
    - Always
    - Mostly
    - Mostly not
    - Never

- Are you involved in planning your work (for example, what is to be done, how it is to be done, or who is to work with you)?
    - Always
    - Mostly
    - Mostly not
    - Never

- How do you experience your work?
    - Likert scale with 5 response items, varying from 1 (too little influence) to 5 (too much influence)

Social support

- Can you receive support and encouragement from your superiors when your work becomes troublesome?
    - Always
    - Mostly
    - Mostly not
    - Never

- Can you receive support and encouragement from your fellow workers when your work becomes troublesome?
    - Always
    - Mostly
    - Mostly not
    - Never
