# Supplementary material for: Development of job demands, decision authority and social support in industries with different gender composition – Sweden, 1991–2013
Source: BMC Public Health. 2019 Jun 14;19:758. doi: 10.1186/s12889-019-6917-8 (PMC6570932; doi:10.1186/s12889-019-6917-8)
Supplement: Supplementary file 3 — Proportion of women exposed to each psychosocial work factor in each wave by industry (PDF 61 kb) [file 12889_2019_6917_MOESM3_ESM.pdf]

Additional file 3. Proportion of women exposed to each psychosocial work factor in each wave by industry

		Edu	HSC	LIS	KIS	PA	GEP	MO
High job demands								
	1991	56.1%	43.5%	48.6%	48.8%	53.0%	36.8%	44.7%
	1993	60.6%	49.3%	47.9%	49.8%	51.6%	43.9%	50.3%
	1995	66.4%	55.8%	50.4%	51.4%	51.9%	41.2%	53.3%
	1997	71.2%	59.9%	55.2%	53.5%	59.2%	47.3%	52.5%
	1999	69.4%	59.8%	60.2%	56.0%	57.0%	52.1%	55.7%
	2001	70.9%	57.0%	51.2%	48.9%	58.1%	43.1%	50.5%
	2003	63.4%	55.1%	52.1%	51.2%	57.6%	46.8%	50.7%
	2005	69.1%	56.1%	54.9%	51.4%	57.4%	45.9%	46.0%
	2007	68.1%	58.5%	55.5%	52.9%	53.2%	52.0%	46.2%
	2009	71.7%	59.2%	51.9%	49.9%	51.0%	43.9%	55.6%
	2011	71.2%	57.9%	50.6%	50.5%	57.4%	47.4%	51.9%
	2013	70.6%	60.6%	52.5%	56.9%	58.7%	52.8%	51.4%
Low decision authority								
	1991	44.1%	53.8%	50.7%	39.9%	38.3%	48.5%	52.8%
	1993	43.7%	55.3%	49.8%	46.2%	36.9%	51.3%	55.5%
	1995	49.7%	58.1%	50.8%	42.7%	35.9%	46.9%	50.7%
	1997	56.2%	62.3%	54.4%	41.6%	38.7%	49.2%	51.2%
	1999	58.9%	62.7%	50.6%	43.0%	40.4%	51.3%	63.8%
	2001	57.0%	63.1%	52.1%	36.2%	41.3%	51.4%	56.4%
	2003	52.5%	59.0%	49.2%	39.7%	38.7%	42.9%	46.4%
	2005	59.7%	63.4%	53.0%	36.6%	44.1%	50.8%	53.1%
	2007	61.5%	62.8%	53.8%	43.8%	42.5%	46.4%	53.5%
	2009	60.1%	63.4%	50.8%	33.8%	41.3%	46.5%	47.3%
	2011	58.4%	60.9%	46.4%	31.3%	43.8%	40.9%	44.6%

2013	60.2%	67.7%	47.4%	40.9%	35.6%	46.9%	43.2%
Poor social support							
1991	32.2%	29.4%	31.1%	34.0%	35.0%	33.1%	35.7%
1993	32.5%	29.8%	34.3%	31.1%	33.7%	34.4%	35.8%
1995	34.2%	34.9%	31.2%	33.6%	37.5%	36.4%	35.7%
1997	38.4%	35.0%	34.2%	34.8%	36.0%	38.5%	34.9%
1999	38.2%	34.5%	35.3%	36.8%	39.5%	35.8%	43.6%
2001	36.8%	37.3%	36.0%	30.6%	37.6%	37.1%	38.1%
2003	35.4%	35.1%	34.8%	35.1%	35.6%	35.6%	31.5%
2005	37.9%	37.2%	41.1%	38.8%	35.1%	41.0%	37.7%
2007	33.9%	37.6%	38.1%	34.6%	31.2%	36.2%	35.2%
2009	35.3%	32.0%	38.2%	30.9%	24.6%	36.1%	36.9%
2011	35.1%	34.4%	36.1%	33.1%	33.0%	28.8%	36.7%
2013	36.6%	36.5%	37.6%	36.5%	33.2%	37.4%	52.6%

Legend:

Edu: Education; HSC: Health and social care; LIS: Labour intensive services; KIS: Knowledge intensive services; PA: Public administration; GEP: Goods and energy production; MO: Machinery operations.

Female-dominated industries: Edu, HSC

Gender mixed industries: LIS, KIS; PA

Male-dominated industries: GEP, MO
