# Supplementary material for: Development of job demands, decision authority and social support in industries with different gender composition – Sweden, 1991–2013
Source: BMC Public Health. 2019 Jun 14;19:758. doi: 10.1186/s12889-019-6917-8 (PMC6570932; doi:10.1186/s12889-019-6917-8)
Supplement: Supplementary file 4 — Proportion of men exposed to each psychosocial work factor in each wave by industry (PDF 61 kb) [file 12889_2019_6917_MOESM4_ESM.pdf]

Additional file 4. Proportion of men exposed to each psychosocial work factor in each wave by industry

	Edu	HSC	LIS	KIS	PA	GEP	MO
High job demands							
1991	58.4%	51.3%	52.6%	52.1%	43.7%	37.1%	42.0%
1993	63.3%	51.0%	55.6%	53.3%	45.6%	46.6%	46.3%
1995	66.1%	52.7%	54.2%	60.2%	49.7%	47.2%	48.9%
1997	66.3%	54.0%	60.8%	59.4%	52.8%	48.3%	51.8%
1999	73.1%	56.8%	57.4%	57.1%	49.0%	47.7%	56.3%
2001	63.9%	53.7%	52.9%	54.6%	48.0%	45.6%	50.5%
2003	57.1%	48.9%	51.1%	51.0%	47.0%	41.9%	51.6%
2005	56.1%	48.2%	51.8%	50.0%	40.7%	47.4%	53.5%
2007	59.7%	46.0%	57.0%	51.1%	47.1%	45.8%	50.4%
2009	63.0%	46.6%	48.8%	49.6%	42.9%	43.9%	46.4%
2011	55.7%	49.0%	49.4%	49.8%	42.8%	42.9%	47.9%
2013	61.1%	54.3%	47.0%	50.3%	42.8%	46.4%	46.8%
Low decision authority							
1991	36.4%	45.7%	30.6%	24.8%	34.1%	41.6%	36.1%
1993	38.9%	44.0%	32.3%	28.9%	33.7%	41.4%	38.3%
1995	35.7%	46.9%	35.6%	24.5%	29.3%	42.7%	38.2%
1997	42.5%	48.5%	35.5%	27.2%	35.4%	48.1%	44.0%
1999	44.7%	57.5%	37.6%	27.2%	40.1%	44.7%	45.0%
2001	42.4%	45.8%	37.5%	26.6%	32.7%	43.5%	43.8%
2003	43.6%	47.4%	35.5%	26.5%	34.6%	42.7%	38.7%
2005	39.6%	45.9%	38.8%	32.9%	32.9%	43.1%	41.6%
2007	39.8%	55.7%	38.6%	26.7%	30.0%	42.7%	40.5%
2009	45.9%	55.0%	35.5%	27.1%	39.3%	41.2%	38.3%
2011	42.1%	54.5%	37.4%	23.8%	30.7%	36.6%	36.1%

2013	44.1%	52.0%	33.9%	24.2%	39.9%	40.5%	39.8%
Poor social support							
1991	46.0%	38.6%	42.2%	46.5%	43.1%	43.5%	44.0%
1993	47.9%	40.6%	46.1%	45.3%	40.4%	44.4%	50.5%
1995	46.4%	42.2%	47.2%	47.3%	38.9%	41.6%	48.1%
1997	49.0%	42.1%	43.9%	38.2%	36.0%	43.2%	47.1%
1999	51.4%	42.9%	41.7%	40.3%	39.3%	41.9%	48.9%
2001	49.4%	42.9%	41.7%	45.2%	43.9%	46.4%	49.1%
2003	46.1%	38.4%	45.2%	40.6%	38.5%	44.1%	43.9%
2005	49.0%	44.1%	49.0%	48.3%	40.4%	46.9%	51.4%
2007	43.8%	42.2%	44.2%	40.1%	37.6%	43.9%	44.9%
2009	41.4%	39.7%	42.0%	40.3%	39.6%	40.3%	45.7%
2011	42.7%	42.0%	39.0%	36.2%	38.2%	38.4%	45.9%
2013	44.6%	39.5%	36.8%	36.3%	36.9%	43.4%	48.0%

Legend:

Edu: Education; HSC: Health and social care; LIS: Labour intensive services; KIS: Knowledge intensive services; PA: Public administration; GEP: Goods and energy production; MO: Machinery operations.

Female-dominated industries: Edu, HSC

Gender mixed industries: LIS, KIS; PA

Male-dominated industries: GEP, MO
